# Lifestyle factors associated with obesity in a cohort of males in the central province of Sri Lanka: a cross-sectional descriptive study

**DOI:** 10.1186/s12889-016-3963-3

**Published:** 2017-01-05

**Authors:** N. W. I. A. Jayawardana, W. A. T. A. Jayalath, W. M. T. Madhujith, U. Ralapanawa, R. S. Jayasekera, S. A. S. B. Alagiyawanna, A. M. K. R. Bandara, N. S. Kalupahana

**Affiliations:** 1 Department of Animal and Food Sciences, Faculty of Agriculture, Rajarata University of Sri Lanka, Anuradhapura, Sri Lanka; 2 Department of Medicine, Faculty of Medicine, University of Peradeniya, Peradeniya, Sri Lanka; 3 Department of Food Science and Technology, Faculty of Agriculture, University of Peradeniya, Peradeniya, Sri Lanka; 4 National Transport Medical Institute, Kandy, Sri Lanka; 5 Department of Agricultural Systems, Faculty of Agriculture, Rajarata University of Sri Lanka, Anuradhapura, Sri Lanka; 6 Department of Physiology, Faculty of Medicine, University of Peradeniya, Peradeniya, Sri Lanka

**Keywords:** Overweight, Obesity, Lifestyle factors, Physical activity, Diet, Sri Lanka, South Asia

## Abstract

**Background:**

Obesity has become a global epidemic. The prevalence of obesity has also increased in the South Asian region in the last decade. However, dietary and lifestyle factors associated with obesity in Sri Lankan adults are unclear. The objective of the current study was to investigate the association of dietary and lifestyle patterns with overweight and obesity in a cohort of males from the Central Province of Sri Lanka.

**Methods:**

A total of 2469 males aged between 16 and 72 years ($\overline{x}=31$) were included in the study. The sample comprised individuals who presented for a routine medical examination at the National Transport Medical Institute, Kandy, Sri Lanka. The Body Mass Index (BMI) cutoff values for Asians were used to categorize the participants into four groups as underweight, normal weight, overweight or obese. The data on dietary and lifestyle patterns such as level of physical activity, smoking, alcohol consumption, sleeping hours and other socio demographic data were obtained using validated self-administered questionnaires. Multinomial logistic regression model was fitted to assess the associations of individual lifestyle patterns with overweight and obesity.

**Results:**

The mean BMI of the study group was 22.7 kg m^−2^ and prevalence rates of overweight and obesity were 31.8 and 12.3%, respectively. Mean waist circumference of the participants was 78.6 cm with 17.1% of them being centrally obese. After adjusting for potential confounders, weight status was associated with older age (*P* < 0.0001), ethnicity (*P* = 0.0033) and higher income (*P* = 0.0006). While higher physical activity showed a trend for being associated with lower odds of being obese (odds ratio: 0.898 – confidence interval: 0.744–1.084), alcohol intake, consumption of fruits, level of education, sleeping hours, smoking, consumption of fish, meat, dairy, sweets or fried snacks were not significantly associated with the weight status.

**Conclusion:**

The high prevalence rates of overweight and obesity in working-age males is a threatening sign for Sri Lanka. Since the prevalence rate is higher in certain ethnic groups and higher-income groups, targeted interventions for these groups may be necessary.

## Background

Obesity has traditionally been considered as a health problem of affluent countries [1], while under nutrition and infectious diseases were considered to be major problems in the developing world [2]. However, with the recent escalation of obesity rates worldwide [3], developing countries, particularly ones in South Asia, are facing a double burden of over and undernutrition [4]. Sri Lanka is a country in South Asia, with a population of more than 20 million. It recently gained the lower-middle-income status. According to the World Health Organization (WHO) non-communicable diseases country profiles, the prevalence rates of overweight (BMI ≥ 25 kg/m^2^) and obesity (BMI ≥ 30 kg/m^2^) among Sri Lankans were 5.1% (2.6% males and 7.4% females) and 21.9% (16.7% males and 26.8% females) respectively in year 2008 [5].

There is a large body of evidence suggesting that the epidemic of overweight and obesity is related to the lifestyle factors of individuals [6–8]. Over time, the relationship between lifestyle patterns and obesity has been extensively studied in western populations, nevertheless, little interest was shown to investigate the risk factors associated with overweight and obesity in South Asia. In fact, limited information is available on different lifestyle patterns associated with overweight and obesity in Sri Lankan adults. Thus, we have been referring to the lifestyle recommendations made for western populations, which is inappropriate since the dietary habits and physical activity patterns of Sri Lankans are different from that of western counterparts [9]. The aim of the present study was to determine the prevalence of overweight and obesity and the underlying lifestyle factors associated with those conditions among a cohort of males in the central Province of Sri Lanka.

## Methods

### Research design and population

A cross-sectional descriptive study was conducted with 2469 adult males aged between 16 and 72 years ($\overline{x}=31$), who presented themselves for a routine medical evaluation done every 4 years at the National Transport Medical Institute, Kandy, Sri Lanka from January 2013 to February 2014. All males who participated in the medical evaluation were considered for the study sample except for the males previously diagnosed with heart diseases, diabetes, hypertension or other chronic illnesses. Institutional review board approval was obtained from the ethics review committee of the Faculty of Medicine, University of Peradeniya, Sri Lanka. (2015/EC/13). All participants in the study signed an informed consent form.

### Data collection

#### Anthropometric measurements

Height, weight and waist circumference (WC) were measured according to the WHO guidelines [10]. The measurements of height to the nearest millimeter and weight to the nearest 100 g were taken using a stadiometer with a scale (Healthweigh^®^ Mechanical Physician Scale (RL-MPS), Goldbell Weigh-System, Singapore). The waist circumference measurement (midpoint between the lowest palpable rib and the superior border of the iliac crest in the mid axillary line at the end of normal expiration) was taken using a non-elastic measuring tape to the nearest millimeter.

The following formula was used to calculate the body mass index (BMI):

$$ \mathrm{B}\mathrm{M}\mathrm{I}\left(\mathrm{kg}/{\mathrm{m}}^2\right)=\frac{\mathrm{Weight}\left(\mathrm{kg}\right)}{\mathrm{Height}\left({\mathrm{m}}^2\right)} $$

BMI cutoff values for Asians defined by WHO [11] were used in the present study to categorize the participants as underweight (BMI <18.5 kg/m^2^), normal (BMI 18.5–22.9 kg/m^2^), overweight (23–27.5 kg/m^2^ and obese (>27.5 kg/m^2^) and named this categorical variable as weight status. Further, central obesity was defined as WC >90 cm for males according to Asian cut-off values [12].

#### Dietary data

A validated, self-administered food frequency questionnaire was used to collect dietary data, where data reflecting the consumption levels of meat, fish, dairy products, fried and salty snacks, sweets and fruits by the participants over the past 6 months (from June 2012 to August 2014) were collected. For the purpose, the participants were asked to provide answers based on their general food consumption patterns and frequency of different foods per week for a period of 6 months.

#### Assessment of the level of physical activity, smoking, alcohol consumption, sleep and socio-demographic data

Physical activity level was assessed using the short version of the International Physical Activity Questionnaire (IPAQ) [13]. Physical activity levels were categorized based on the number of minutes they had participated in moderate-intensity and/or vigorous-intensity activity during the week. When a person participated in less than 150 min of moderate-intensity physical activity or less than 75 min of vigorous-intensity activity per week, it was considered as low physical activity level whereas participation in 150–300 min of moderate-intensity activity or 75–150 min of vigorous-intensity physical activity per week was considered as medium physical activity level. A person was considered to have a high physical activity level when that person participated > 300 min of moderate-intensity physical activity per week [13–15]. Smoking, alcohol consumption and duration of sleep were assessed using a self-administered questionnaire.

Data on age, gender, ethnicity, level of education and household income were collected using an interviewer-administered questionnaire. Educational level was classified into four categories: no formal education to primary education (grade 1–5), secondary education–1 (grade 6–11) secondary education–2 (grade 12–13) and tertiary education (under-/post-graduate) [adopted and modified from 16]. Monthly household income was categorized as follows: Sri Lankan rupees (LKR) < 6999, LKR 7000–12 999, LKR 13 000–24 999, LKR 25 000–49 999 and > LKR 50 000 [16]. (1 USD = 145 LKR).

Smoking score was developed based on the number of cigarettes smoked per day by each individual. When a person smoked 1–10 cigarettes per day, that person was considered as a moderate smoker while > 10 cigarettes per day, a heavy smoker [17]. The number of hours slept per day by each individual was used to construct the sleeping score. When a person slept for < 6 h per day that was considered as a low sleeping score. Medium sleeping score was considered when a person slept for 7–8 h per day while > 8 h of sleep per day was considered a high sleeping score [18].

### Statistical analysis

Data were analyzed using SAS 9.3 (SAS Institute Inc., Cary, NC). Descriptive statistics such as mean and Standard Deviation (SD) were computed for continuous variables and frequencies and percentages were computed for categorical variables. Since the dependent variable, weight status, has four categories, we performed multinomial logistic regression [19] to estimate odds ratios (ORs), considering normal weight as the reference category. Hence, the associations of dietary variables and other lifestyle variables on weight status were assessed using a single model. In this model, independent variables, alcohol intake, sleeping hours, smoking, consumption of fruits, fish, meat, dairy, sweets and fried snacks considered as numerical variables and entered into the model as frequency per week. Further, age was considered a numerical variable and other independent variables (education, income category and ethnicity) were entered into the model as categorical variables. Effect of each variable was tested after adjusting for other confounding variables. A significant level of 0.05 was considered.

## Results

Baseline characteristics of the study variables are summarized in Table 1 and demographic characteristics of the study sample are summarized in Table 2. Mean age of the study sample was 31 years with a mean BMI of 22.7 kg/m^2^. Mean WC was 78.6 cm and 17.1% of the study sample were centrally obese (Table 3).

**Table 1:** Baseline characteristics of the study sample

Characteristic	Mean	± SD
Age (years)	31	10.27
Height (m)	1.65	0.059
Weight (kg)	62.54	11.96
BMI (kg/m^2^)	22.71	4.22
WC (cm)	78.67	11.33

*n* = 2466; *BMI* body mass index, *SD* standard deviation, *WC* waist circumference

**Table 2:** Socio-demographic characteristics of the study group

	Number	% of participants
Age category (*n* = 2466)		
< 30	396	16.06
31–40	816	33.09
41–50	717	29.08
51–60	415	16.83
> 60	122	4.95
Ethnicity (*n* = 2461)		
Sinhala	2065	83.91
Tamil	191	7.76
Moor	201	8.17
Other	04	0.16
Level of education (*n* = 2342)		
No education – grade 5	17	0.73
Grade 6 – grade 11	353	15.07
Ordinary level passed	1229	52.48
Advanced level passed	679	28.99
Graduate/postgraduate	64	2.73
Monthly household income (*n* = 1830)		
≤ LKR 6999	31	1.69
LKR 7000–12,999	151	8.25
LKR 13,000–24,999	832	45.46
LKR 25,000–49,999	720	39.34
≥ LKR 50,000	96	5.25
Smoking score (*n* = 2466)		
Non smoker	2112	85.64
< 10 cigarettes per day	349	14.15
> 10 cigarettes per day	5	0.20
Alcohol consumption (*n* = 2464)		
Non alcoholic	1902	77.19
Alcoholic	554	22.48
Sleeping score (*n* = 2466)		
≤ 6 h/day	93	3.77
7–8 h/day	1939	78.63
> 8 h/day	394	15.98
Physical activity level (*n* = 2466)		
Low	465	18.86
Medium	345	13.99
High	1590	64.48

LKR–Sri Lankan Rupees (1USD = 145 LKR)

**Table 3:** Waist circumference levels (95% Confidence interval (CI)) of the study population

Waist circumference level	% (CI)	Risk level	Central obesity
< 90 cm	82.89 (81.48, 84.39)	No risk	None
90–102 cm	14.4 (12.96, 15.87)	High risk of developing NCDs	Present
> 102 cm	2.72 (1.30, 4.21)	Greater risk of developing NCDs	Present

Within the study sample, 22.48% consumed alcohol and 14.35% were smokers where 14.15% of them were moderate smokers while only 0.2% of them were heavy smokers. Majority of the participants in the study sample (78.63%) had 7–8 h of sleep per day. Self-reported physical activity levels revealed that 64.48% of the study sample had high a physical activity level of 300 min of moderate-intensity physical activity per week. Nearly 99% of the participants in the study sample had received school education, while nearly 40% of the sample had a fairly good income (approx. US$ 250–450).

**Fig. 1:**
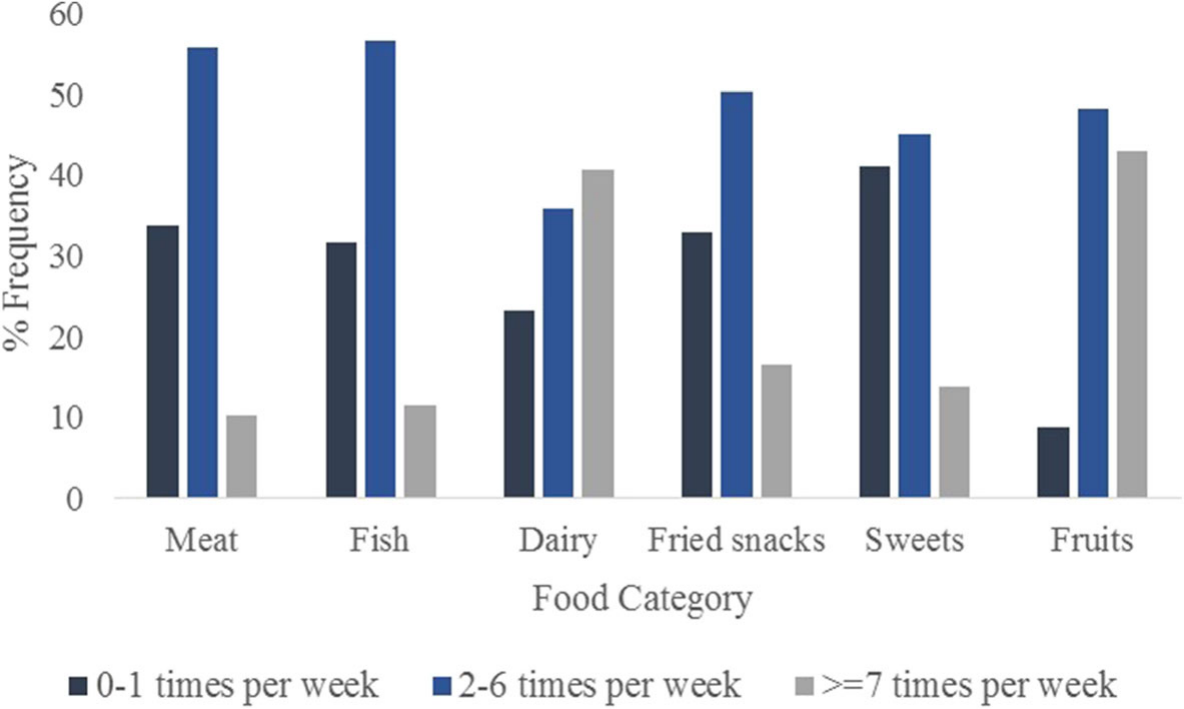
Frequency of different food consumption of the study sample. Intake of different foods were assessed using a self-administered food frequency questionnaire

Self-reported frequency of meat, fish, dairy, fried snacks, sweets and fruits consumption of all participants in the study sample is shown in the Fig. 1. Results revealed that, 28% of the participants in the study sample consumed fruits at least seven times per week whereas only 14.5% of the study sample consumed more than one portion of fruits per day (all together 42.5% consumed one or more fruit per day–Fig. 1).

### Prevalence of overweight and obesity

The prevalence rates of overweight and obesity were 31.8 and 12.3%, respectively (Table 4). Overweight and obesity were higher among males aged between 41 and 50 years compared to the younger age groups. According to the results, when age increased by 10 years, the males were more likely to be overweight (OR: 1.449) or obese (OR: 1.647) than being normal weight persons. The oldest age group (age >60 years) had the highest levels of overweight (43.44%) and obesity (22.95%). The results also showed that, Moors were more prone to be overweight (OR: 1.684) or obese (OR: 2.608) than Sinhalese. Moreover, the odds of being overweight was higher for income groups 4 (OR: 2.742) and 5 (OR: 3.305) compared to income group 1 (Table 5). Table 6 gives the odds ratios and confidence interval of significant variables for overweight and obese compared to normal weight group. Among the variables studied, age, ethnicity and family income were significantly (*P* < 0.05) associated with weight status. When the level of physical activity was considered, higher physical activity showed a trend for being associated with lower odds of being obese (odds ratio: 0.898 – confidence interval: 0.744–1.084) (Table 6). Alcohol intake (*P* = 0.058), level of education (*P* = 0.1246), sleeping hours (*P* = 0.9847), smoking (*P* = 0.5872), consumption of fish (*P* = 0.6042), meat (*P* = 0.7729), dairy (*P* = 0.6190), fruits (*P* = 0.1803), sweets (*P* = 0.4472) and fried snacks (*P* = 0.8792) were not significantly associated with weight status.

**Table 4:** Prevalence (95% CI) of overweight and obesity according to BMI cut-offs for Asians

	Percent	CI
Overweight	31.8	29.68, 33.93
Obese	12.3	10.22, 14.46

*CI* confidence interval

**Table 5:** Prevalence (95% CI) of overweight and obesity among males by age, ethnicity, income category and education level

Variable	Overweight % (CI)	Obese % (CI)
Age category (*n* = 2466)		
< 30	16.16 (11.11, 21.48)	7.58 (2.53, 12.89)
31–40	23.90 (20.34, 27.59)	9.07 (5.51, 12.76)
41–50	42.12 (38.35, 46.16)	14.23 (10.46, 18.27)
51–60	40.96 (35.90, 46.15)	16.87 (11.81, 22.05)
> 60	43.44 (34.43, 53.18)	22.95 (13.93, 32.69)
Ethnicity (*n* = 2461)		
Sinhalese	31.67 (29.39, 34.02)	11.91 (9.64, 14.26)
Tamil	32.98 (25.65, 40.60)	12.04 (4.71, 19.66)
Moor	32.84 (25.87, 40.43)	15.92 (8.96, 23.52)
Income category (*n* = 1830)		
≤ LKR 6999	22.58 (6.45, 41.11)	9.68 (0.00, 28.20)
LKR 7000–12,999	28.48 (20.53, 37.13)	13.91 (5.96, 22.56)
LKR 13,000–24,999	34.13 (30.53, 37.80)	9.86 (6.25, 13.52)
LKR 25,000–49,999	39.17 (35.28, 43.09)	16.11 (12.22, 20.03)
≥ LKR 50,000	43.75 (33.33, 54.22)	20.83 (10.42, 31.31)
Education level (*n* = 2342)		
No education – grade 5	23.53 (5.88, 50.64)	11.76 (0.00, 38.87)
Grade 6 – grade 11	30.59 (25.21, 36.21)	11.90 (6.52, 17.51)
Ordinary level passed	33.41 (30.48, 36.49)	11.65 (8.72, 14.73)
Advanced level passed	30.97 (26.99,35.00)	14.75 (10.77, 18.78)
Graduate/postgraduate	31.25 (20.31, 45.23)	14.06 (3.13, 28.05)

*CI* confidence interval

**Table 6:** Odds ratios of overweight and obesity in males – multinomial logistic regression analysis

Covariate	Overweight OR (95% CI)	Obesity OR (95% CI)	*P* value
Age	1.449 (1.280, 1.641)	1.647 (1.391, 1.950)	< 0.0001
Ethnicity			0.0023
Moor vs Sinhalese	1.684 (1.067, 2.658)	2.608 (1.492, 4.560)	
Tamil vs Sinhalese	1.513 (0.990, 2.312)	1.726 (0.981, 3.036	
Family income			0.0164
Group 5 vs group 1	3.305 (1.105, 9.890)	2.906 (0.703, 12.107)	
Group 4 vs group 1	2.742 (1.023, 7.348)	2.278 (0.424, 8.321)	
Group 3 vs group 1	2.356 (0.882, 6.290)	1.441 (0.394, 5.266)	
Group 2 vs group 1	1.739 (0.608, 4.975)	1.990 (0.506, 7.826)	
Physical activity level	1.138 (0.983, 1.317)	0.898 (0.744, 1.084)	0.0122
High vs low	1.302 (0.965, 1.788)	0.836 (0.564, 1.238)	
Medium vs low	1.108 (0.739, 1.682)	1.244 (0.788, 2.044)	

*n* = 1689

## Discussion

Obesity is an emerging problem in the South Asian region. However, the lifestyle factors associated with obesity in this region are not well studied. This knowledge is required to design tailor-made interventions to prevent obesity. Thus, the purpose of this study was to identify lifestyle factors associated with obesity in a cohort of males in the Central Province of Sri Lanka. The prevalence rates of overweight and obesity in this group were 31.8 and 12.3%, respectively, with the prevalence rate of central obesity being 17.1%.

In this study, the mean BMI and WC reported were 22.7 kg/m^2^ and 78.67 cm, respectively. Similar mean BMI (21.1 kg/m^2^) and WC (78.0 cm) for males were reported in a national study conducted by Katulanda et al. [20] which was carried out in seven provinces of Sri Lanka in 2010. Fairly comparable BMI and WC values were observed among few Asian male populations: India 22.6 kg m^−2^ [21]; 85.6 cm [22], Korea 23.2 kg m^−2^ [23]; 84.3 cm [24], Pakistan 20.9 kg m^−2^; 77.7 cm [25] and Bangladesh 19.3 kg m^−2^ [26]; 72.8 cm [27]. Between 1980 and 2008, mean BMI of males worldwide increased by 0 · 4 kg/m^2^ per decade [28]. Simultaneously, the mean BMI of the Sri Lankan rural and urban population has increased significantly during the past decade possibly due to nutrition transition [20]. In addition to sedentary lifestyle and poor dietary habits, negative effects of globalization, urbanization, and increasing age of the adult population likely contributed to this increasing BMI [29].

Current study revealed that the prevalence rates of overweight and obesity among men in the Central Province of Sri Lanka were 31.8 and 12.3%, respectively based on the WHO cut-off values for Asians (Table 4). However, Katulanda et al. [20] reported that 25.2% of the adult Sri Lankan population were overweight, while 9.2% were obese in the year 2010, which are lower compared to the findings of the present study. Further, findings of the study conducted in 2010 by Wijewardana et al. [30], reported a prevalence rate of overweight or obesity in males in four provinces of Sri Lanka as 20.3%, reflecting a trend of increasing obesity, as seen in many countries. Nevertheless, this is much lower than the prevalence rates of overweight (BMI ≥ 25.0 kgm^−2^–66.3%) and obesity (BMI ≥ 30.0 kgm^−2^–32.2%) in males in the USA in 2003/2004 [31]. Asian countries are also showing an increasing trend of overweight and obesity [32]. Asian region contains some of the most populous countries in the world (China and India), and has under gone pronounced demographic, epidemiologic, and socio economic change in recent decades. In China, the prevalence rate of overweight (≥ 25 · 0 kg/m^2^) and obesity (≥ 30 · 0 kg/m^2^) were 25.5 and 4.7% in 2008 in men respectively whereas in India they were 9.9 and 1.3% respectively in 2008 in men [5].

According to Katulanda et al. [20] female sex, living in urban environments, a high level of education, high income and being in the middle age were the risk factors for overweight and obesity in Sri Lankan adults. Present results indicate that among the variables studied, increased age, ethnicity, high family income and low physical activity level (trend) are associated with overweight and obesity. It was discovered that when the age increases by 10 years, a person is more likely to become overweight or obese. The present study further observed that, individuals aged 31–50 years had significantly higher risk for being obese than individuals less than or equal to 30 years. These results are comparable with the findings of Marengoni et al. [33], that increasing age was associated with a more than 50% increased risk for multi-morbidity. Similar findings have also been observed by several research studies [21, 34, 35], where aging is considered as a risk factor for becoming obese.

Present data also revealed that there are ethnic differences in the prevalence rates of overweight and obesity. Moors showed higher incidences of overweight and obesity compared to Sinhalese. This may be due to the different dietary habits associated with diverse ethnic groups. Similar to the present findings, De Silva et al. also observed a higher prevalence rate of obesity in Moor community in their research conducted in Kalutara district of Sri Lanka [36]. This observation is also supported by the findings of the research carried out by Katulanda et al. [37] among Sri Lankan adults in 2012, where they have found out that Moors were more physically inactive than Tamils and Sinhalese which was associated with obesity and other chronic diseases such as cardiovascular diseases, diabetes and hypertension. Further, in 2014, Jayawardena et al. reported that Moors have a higher energy and protein intake and consume more fat rich food compared to Indian Tamils, Sri Lankan Tamils and Sinhalese [38]. However, ethnic difference was not recognized in the two large surveys conducted in Sri Lanka in years 2005 and 2006 on obesity [20, 30].

Jayawardena et al. [39] reported that daily intake of fruits and dairy among Sri Lankans (only 0.4 portions/day) are well below the national recommendations (2–3 portions/day), and the dietary pattern of the present study population reflected that the consumption of fruits was indeed low. Many studies reported an inverse relationship between consumption of fruits and weight gain [40–43] while few studies reported no association between increased consumption of fruits and weight gain [44–46]. Our study did not find a significant association between fruit intake and obesity, maybe due to the low level of fruit intake in the sample. In contrast to previous cross sectional studies showing a positive association between alcohol intake and BMI [47–49], the current study showed a trend for alcohol consumption to have a negative association with overweight and obesity. Similar results were reported by several research studies [50–52]. Further, a 9 year follow up study done by Wang et al. with 19,220 women also showed that a higher alcohol intake at baseline was associated with a lower risk of becoming overweight or obese in the following years [53]. Two other cohort studies found no significant association between alcohol intake and BMI [54, 55]. In addition, two research studies have confirmed that obesity was inversely associated with drinking frequency [56, 57]. This may due to the fact that drinkers usually substitute alcohol for other foods [53, 56, 57] potentially leading to a negative energy balance.

Some studies suggested that number of sleeping hours have positive relationship with obesity [19, 58] while others suggest that less sleeping hours increase the incidences of obesity [59, 60]. However, this study revealed that sleeping hours did not have any relationship with overweight or obesity which is reported similarly in a clinical review done by Marshall et al., where they have found out that neither long nor short sleep was associated with obesity [61].

Low levels of physical activity has been shown to be associated with increased obesity in many research studies conducted worldwide including Sri Lanka [18, 20, 62, 63]. Further, there is an inverse association of high physical activity with obesity and unhealthy weight gain [64–66]. We did find a trend for higher physical activity to associate with lower odds of being obese (odds ratio: 0.898 – confidence interval: 0.744–1.084).

We found out that overweight and obesity were common among men with higher income levels. Similar to our findings, Katulanda et al. and De Silva et al. found a positive association between obesity and increasing income levels in Sri Lankan adults [20, 67]. India and Bangladesh similarly show an increase in obesity prevalence rates with increase in education levels and living standards [68, 69]. This may be attributed to nutrition transition, with increased availability of food as well as money to purchase food, which will increase energy intake leading to obesity. However, this is opposite in higher income countries, where higher prevalence of obesity is seen in low socio economic strata [70, 71]. Nonetheless, a review by Monteiro et al. in 2004 stated that the burden of obesity in developing countries shifts to low socio economic groups, when the country’s gross national product increases [72].

The current study has a few limitations. We collected data from people in the Central province, who presented themselves for a medical evaluation at the National Transport Medical Institute, Kandy, Sri Lanka. Since more than 90% of this sample were males, we only included data about males in this study. Further, due to limitations in human resource to conduct the survey, we had to conduct self-administered questionnaire which is less effective than interviewer administered questionnaires.

## Conclusion

High prevalence of overweight and obesity in working age males is a threatening sign for Sri Lanka. Obesity in Central province is higher among high socio economic groups and in the Moor community. It is also evident that obesity prevalence represents a public health problem as it increases the economic burden and health risk factors of the community. As this population ages in the future and urbanization continues, the prevalence of overweight and obesity will likely to escalate. This will result in an aging population burdened with obesity as well as its deleterious effects such as cardiovascular disease, type 2 diabetes, hypertension and bone and joint disease. Since the prevalence rate is higher in certain ethnic groups and higher-income groups, targeted interventions for these groups may be necessary.

## Abbreviations

BMI: Body mass index
CI: Confidence interval
IPAQ: International Physical Activity Questionnaire
LKR: Sri Lankan rupees
OR: Odds ratio
SD: Standard Deviation
WC: Waist circumference
WHO: World Health Organization
